# Machine Learning-Based Classification of Lignocellulosic Biomass from Pyrolysis-Molecular Beam Mass Spectrometry Data

**DOI:** 10.3390/ijms22084107

**Published:** 2021-04-15

**Authors:** Ambarish Nag, Alida Gerritsen, Crissa Doeppke, Anne E. Harman-Ware

**Affiliations:** 1 Computational Science Center, National Renewable Energy Laboratory, 15013 Denver West Pkwy, Golden, CO 80401, USA; ambarish.nag@nrel.gov (A.N.); alida.gerritsen@nrel.gov (A.G.); 2 Renewable Resources and Enabling Sciences Center, National Renewable Energy Laboratory, 15013 Denver West Pkwy, Golden, CO 80401, USA; crissa.doeppke@nrel.gov

**Keywords:** biomass analysis, pyrolysis, molecular beam mass spectrometry, classifiers, random forest, decision tree, Gaussian Naïve Bayes, gradient boosting, multilayer perceptron

## Abstract

High-throughput analysis of biomass is necessary to ensure consistent and uniform feedstocks for agricultural and bioenergy applications and is needed to inform genomics and systems biology models. Pyrolysis followed by mass spectrometry such as molecular beam mass spectrometry (py-MBMS) analyses are becoming increasingly popular for the rapid analysis of biomass cell wall composition and typically require the use of different data analysis tools depending on the need and application. Here, the authors report the py-MBMS analysis of several types of lignocellulosic biomass to gain an understanding of spectral patterns and variation with associated biomass composition and use machine learning approaches to classify, differentiate, and predict biomass types on the basis of py-MBMS spectra. Py-MBMS spectra were also corrected for instrumental variance using generalized linear modeling (GLM) based on the use of select ions relative abundances as spike-in controls. Machine learning classification algorithms e.g., random forest, k-nearest neighbor, decision tree, Gaussian Naïve Bayes, gradient boosting, and multilayer perceptron classifiers were used. The k-nearest neighbors (k-NN) classifier generally performed the best for classifications using raw spectral data, and the decision tree classifier performed the worst. After normalization of spectra to account for instrumental variance, all the classifiers had comparable and generally acceptable performance for predicting the biomass types, although the k-NN and decision tree classifiers were not as accurate for prediction of specific sample types. Gaussian Naïve Bayes (GNB) and extreme gradient boosting (XGB) classifiers performed better than the k-NN and the decision tree classifiers for the prediction of biomass mixtures. The data analysis workflow reported here could be applied and extended for comparison of biomass samples of varying types, species, phenotypes, and/or genotypes or subjected to different treatments, environments, etc. to further elucidate the sources of spectral variance, patterns, and to infer compositional information based on spectral analysis, particularly for analysis of data without a priori knowledge of the feedstock composition or identity.

## 1. Introduction

The thermal decomposition of lignocellulosic biomass in the absence of oxygen, known as pyrolysis, has been used to study the composition and structure of the cell walls at an analytical scale and also to convert the biomass to liquids and gases for larger scale chemical production. Pyrolysis coupled with molecular beam mass spectrometry (py-MBMS) can be used to rapidly analyze pyrolysates and corresponding ions generated from the decomposition of different biopolymers present in biomass based on the abundance of various ions in the resulting spectra. Many studies have used high-throughput py-MBMS to screen and study changes in lignin content and composition, as well as sugar and terpenoid composition of lignocellulosic biomass [1,2,3,4,5,6,7,8,9]. Additionally, the source of many pyrolysates and their corresponding ions and fragmentation patterns have also been thoroughly investigated using various pyrolysis-mass spectrometry systems [1,2,8,10,11,12,13]. Unfortunately, the spectra obtained from py-MBMS are not easily deconvoluted, as they are collected without chromatographic separation in addition to the confounding of variables whereby many ions could come from numerous pyrolysates, which could in turn originate from different sources in the feedstocks. Additionally, spectral features may not necessarily be additive or quantitatively linear, and the pyrolysis process and other biomass properties could impact the relative abundance of different ions [14,15,16]. While previous studies have used multivariate approaches and basic regression techniques to predict biomass composition on the basis of py-MBMS spectra, the majority of work has focused on a small fraction of ions of known origin present in the spectra [1,2]. Additionally, many other computational and statistical tools are available and being used to study pyrolysis mass spectrometry data as it relates to lignocellulosic biomass composition and genomics, but there is currently a lack of information regarding modern and consistent data analytics pipelines for comprehensive spectral analysis and comparison [5,17,18].

Many basic statistical data analysis tools and algorithms can be used to interpret and reduce the number of necessary features or dimensionality of py-MBMS spectra of lignocellulosic biomass and other biological feedstocks such as fungi and bacteria. Multivariate data analysis, factor and discriminant analysis (DA), and principal component analysis (PCA) have been used to explain spectral patterns and variation as well as make classifications and predictions primarily on the basis of feedstock composition [1,2,10]. Supervised learning, regression analyses, and other methods such as partial least squares (PLS), principal component regression (PCR), and artificial neural networks (ANN) have been used to predict compositional or product data generated from py-MBMS analyses of feedstocks such as cattle manure [19], *E. coli,* and other microorganisms [20,21,22,23]. Other examples of the use of ANN and random forests (RF) for various applications using mass spectral data, particularly for microorganisms, are explored in additional works by Goodacre et al. [23,24,25].

Workman Jr. and Howard [26] recently surveyed chemometric methods used in the field of spectroscopy. These methods can be roughly divided into four groups. The first group includes signal preprocessing techniques (e.g., baseline subtraction, normalization, and standard normal variate) that are often used prior to the application of explorative, qualitative, or quantitative methods. The second group consists of component analysis techniques used mostly for data exploration and discovery, e.g., independent component analysis, multi-variate curve resolution, and principal component analysis. The third group comprises a variety of quantitative (calibration) methods used to compute predictive calibration models for the quantitative determination of physical or chemical parameters in a dataset from raw or preprocessed data. Examples of methods in the third group include multiple linear regression, partial least squares regression, principal component regression, and ANNs. The fourth group encompasses qualitative (calibration) methods used to take raw or preprocessed data and compute predictive calibration models for qualitative (classification) of different groups or types of samples or of physical or chemical parameters in a dataset. Some of the members of this class of chemometric methods are common machine learning algorithms for classification, e.g., the k-nearest neighbors (k-NN) classifier, logistic regression, ANNs, random forest classifier, decision tree classifier, naïve Bayesian classifier, etc. We have used selected methods from all four categories described above for the analysis of py-MBMS spectral data in the current work as described in detail in the later sections of this paper.

It is essential that modern data analysis methods be developed and optimized for the rapid analysis of biomass feedstocks to inform systems biology models and aid in analysis points across the bio-derived value chain. The availability and utilization of proper data analysis tools for coupling high throughput pyrolysis and compositional information can enable the understanding of biomass growth and cultivation as well as processes and conversion routes used to generate bio-derived products. The goal of this manuscript is to develop a workflow for the classification of lignocellulosic biomass on the basis of py-MBMS spectra. Here, we outline a workflow for analyzing large lignocellulosic datasets from py-MBMS that can be extended to studies incorporating feedstocks of one or more species for which the pyrolysis analysis is used to elucidate compositional and structural variation in cell wall components. To this end, we have compared common machine learning classification algorithms to determine which algorithm(s) work best for the classification of biomass using py-MBMS data. This manuscript outlines methods that can be used to elucidate spectral variation to provide clues regarding inherent differences in the samples without a priori knowledge of the sample composition or when similar standards are not available. This method can also be extended and used for rapid biomass analysis and screening purposes.

## 2. Results

### 2.1. Sourcing and Removal of Instrumental Variance

The py-MBMS datasets were first processed in the MBMS software (Merlin V 3, Extrel CMS) to generate an average spectrum across total ion chromatograms for each biomass peak such that each biomass sample yielded one spectrum for further data analysis. Then, each biomass spectrum was normalized to the total ion intensity or separately corrected for instrumental variance using principles of RNA sequencing analysis that are typically used to control technical variance across instruments with control samples [27]. Then, the corrected or normalized spectra were termed “RUV” spectra, and each RUV corrected spectrum was then subsequently normalized to the total ion intensity. The underlying assumptions of normalizing large datasets across different instruments for RNAseq are applicable to py-MBMS analysis: (1) data are complex and contain replicates; (2) there are differences in sample loadings; (3) occurrence of operator technician variation. Normalization strategies for py-MBMS have traditionally included reference standards from NIST and internal controls, but they typically fail to account for instrument drift across time or space and thus can only be comparable within runs, although we have previously reported one different type of correction or normalization strategy [28]. Similar limitations occur across RNA sequencing runs, which vary in library depth, preparation, and reagent quality. In order to be able to compare pyrolysis data generated at different times, we have used the component ions of S/G/L (syringyl/guaiacyl/lignin) present in control (Aspen) samples that are not expected to vary substantially with technical differences in sample preparation and hence serve as “internal standard” metrics that can be used as reference values. Following the methods detailed for RUVg in [27], we adjusted the spectral data to remove the variation due to instrument drift and compared the py-MBMS results and data analysis as shown in further sections.

### 2.2. Statistics, Principal Component Analysis, and Clustering

The ion intensities in the spectra were normalized to the sum of the ion intensities for each spectrum. Next, to aid in efforts to classify or characterize the lignocellulosic biomass composition based on the spectra, we also performed statistical analyses including principal component analysis, hierarchical clustering, and basic descriptive statistics (means, ranges, standard deviations, Pearson correlations, etc.). A priori classification and compositional information (Table 1) of the standard biomass set was used to build classification models described later. When relevant standards or models are not available to predict characteristics, the statistical and principal component analyses may be used to predict relative compositional or structural variation with some degree of certainty when used in conjunction with known ion annotations.

**Figure 1:**
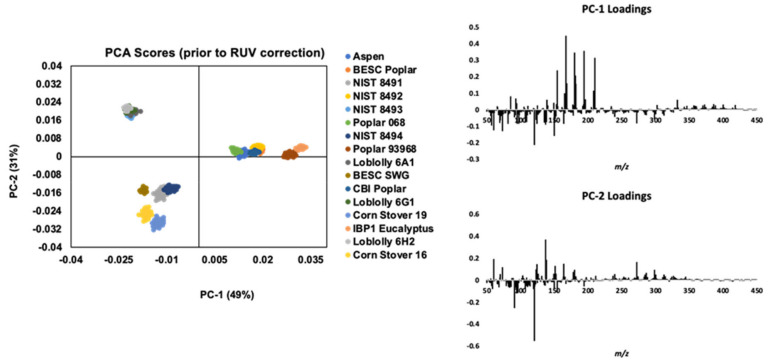
Principal component analysis of pyrolysis followed by mass spectrometry such as molecular beam mass spectrometry (py-MBMS) data from different types of lignocellulosic biomass.

Statistical analyses for the biomass experimental datasets are provided in the Supplementary Information File S1, SIFile1_DescStats.xlsx. The ions that varied to the greatest degree (largest range) across the different biomass types were generally derived from lignin and sugars corresponding with the range in abundance and structure of these biopolymers in the biomass. Principal component analysis of the biomass standard samples is outlined in Figure 1. The first principal component explained 49% of the variation (43% with removal of unwanted variance (RUV) instrumental drift correction), and the second principal component explained 31% of the variation (37% RUV corrected) in the different biomass type dataset. The loadings plotted as spectra demonstrate a general negative correlation between lignin-derived peaks (*m*/*z* 154, 167, 180, 194, 208, 210) and sugar-derived peaks (*m*/*z* 84, 95, 96, 97, 114) (also see Supplementary Information File S2 SIFile2_PearsonCC.xlsx for Pearson correlation coefficients for RUV/instrumental drift corrected ions). However, ions such as 120 and 150, which may derive from coumarates and ferulates, respectively, and may also derive from lignins, do appear to negatively correlate with other lignin peaks. Additionally, some ions did positively correlate with lignin peaks that arise derive from sugars (such as *m*/*z* 126).

As shown in the PCA scores plot in Figure 1 and similarly to that reported previously [1,2,7,9,28], samples cluster according to primary biomass type (family, being hardwood, softwood, or grasses) based on their spectra prior to RUV correction (instrument drift correction) from *m*/*z* 30–450 and to a lesser degree, secondary biomass type (species) and Sample ID. The first principal component (x-axis), as outlined before, generally separates the biomass types according to relative lignin and sugar abundance. The second principal component (y-axis) separates the biomass according to the apparent negative correlation (Pearson correlation = −0.61, Supplementary File S2) between *m*/*z* 120 (coumaryl and/or coumarate-derived) and 137 (guaiacyl lignin-derived) spectral peaks.

The removal of instrument drift in the spectra by RUV correction resulted in similar loadings patterns for the first and second principal components (see Supplementary File S3 SIFile3_PCARUVFigure.pdf). The ions of highest abundance originating from the primary components of plant cell walls (particularly lignin) were still the main drivers of variation (PC-1 Loadings, Figure 1 and Supplementary Files S2 and S3) where lignin-derived ions (*m*/*z* 124, 137, 154, 167, 180, 194, 210) were generally negatively correlated with ions derived from ferulate (150) and coumarate (120), which are generally more abundant in grasses than in hardwoods and softwoods. Differences are noted in the principal components (PC-1 loadings) prior to RUV where the ions derived from lignin S (such as 154, 194, 210) and G monomers (124, 137) were projected negatively, whereas after RUV correction they were projected on the same axis. In addition, as expected, ions were disproportionately affected by the RUV correction and PC-2 loadings differed significantly before and after RUV correction. Prior to RUV correction, PC-2 loadings are primarily driven by G-lignin-derived ions and negatively correlated coumarate and ferulate-derived ions (120, 150), whereas the RUV corrected PC-2 loadings show negative correlation between lignin S (such as 154, 194, 210), and G monomers (124, 137) and *m*/*z* 120 loading is insignificant.

### 2.3. Classification Models

#### 2.3.1. Classification of Biomass Prior to Removal of Unwanted Variance (RUV) Correction

The results from the machine-learning based prediction models before the removal of instrumental variance from the classification of spectral data of the biomass standards set are based on the classifications outlined in Table 1. In the current study, there are three possible biomass feedstock primary types or families: hardwoods, softwoods, and grasses. There are 10 possible biomass feedstock secondary types: *Poplulus tremuloides*, *Populus trichocharpa*, Sugarcane Bagasse, *Poplulus deltoides*, Monterey Pine, Wheat Straw, Loblolly Pine, Switchgrass (*Panicum virgatum*), Corn Stover, and Eucalyptus. The 16 possible Sample IDs for biomass feedstocks are Aspen (*Poplulus tremuloides*), BESC Poplar (*Populus trichocharpa)*, NIST 8491 (Sugarcane Bagasse), NIST 8492 (*Poplulus deltoides*), NIST 8493 (Monterey Pine), Pop 068 (*Populus trichocharpa)*, NIST 8494 (Wheat Straw), Pop 93968 (*Populus trichocharpa)*, Lob 6A1 (Loblolly Pine), BESC SWG (*Panicum virgatum*), CBI Poplar (*Populus trichocharpa)*, Lob 6G1 (Loblolly Pine), Corn Stover 19, IBP1 (Eucalyptus), Lob 6H2 (Loblolly Pine), and Corn Stover 16.

A total of 816 individual samples were analyzed where 127 *m*/*z* spectral intensities were selected as the largest comprehensive set of important features within each spectrum. These 127 *m*/*z* spectral intensities were chosen based on spectral peak annotations from previous work and domain knowledge. These *m*/*z* spectral peaks correspond to carbohydrate-derived pyrolysates, different lignin monomers, and other components such as ferulates and coumarates [8,28,29,30]. The removal of correlated features resulted in 106 instead of 127 features, whereas using *p*-value-based feature selection lowered the number of features to 48, 47, and 43 for the prediction of primary type, secondary type, and Sample IDs, respectively.

Table 2 provides classification results obtained for specific values of random number seeds that are kept invariant between the different machine learning (ML) classifiers to make the results reproducible. It is evident from Table 2 that although all the classifiers have comparable performance, the k-NN classifier performs the best for all three classifications, whereas the decision tree classifier performs the worst. However, it should be noted that for the given number of data points, all ML classifiers perform equally well for predicting the three primary types for both the complete and reduced feature sets. On the other hand, the prediction accuracies of the secondary type and Sample IDs, which have more categories, display some variation between the different ML algorithms for classification and for the different feature sets used.

Details about the features corresponding to the full feature set of 127 spectral intensities for all the three different classification problems are provided in the Supplementary Information File S4 SIFile4_Features_all.xlsx. The subset of 106 non-correlated spectral intensities, including the importance of each feature for the different ML algorithms used for the primary type, secondary type, and Sample ID classification problems are described in the Supplementary Information File S5 SIFile5_Features_corr.xlsx. The smaller subsets of features obtained using *p*-value for all the three classifications are described in the Supplementary Information File S6 SIFile6_Features_pcorr.xlsx.

It is evident from SIFile4_Features_all.xlsx that in the case of predicting the primary type using all 127 spectral features, the two most equally important features for five of the six ML algorithms used were the spectral intensities at the *m*/*z* values of 167 and 194. Both of these peaks derive primarily from syringyl ligin [7,8,29]. The spectral intensities at 91, 107, 137, 154, 167, 168, 181, 194, 196, 208, and 210 *m*/*z* values frequently occurred in the top 10 features for all or most of the six different types of ML algorithms used for predicting the primary type. The corresponding ions derive from a variety of phenolics including syringyl and guaiacyl-derived lignin monomeric units [28,29].

For prediction of the secondary type using all 127 spectral features, there was more variation between the different ML algorithms with respect to the most important spectral feature. For three of the six ML algorithms used, the most important feature was the spectral intensity at the *m*/*z* value of 194 (corresponding to the production of 2,6-dimethoxy-4-(2-propenyl)phenol from pyrolysis of syringyl lignin bound by labile linkages); for two others, the most important feature was the spectral intensity at the *m*/*z* value of 168 (derived from 4-methylsyringol, but potentially bound by different linkages than syringyl monomers due to the fragmentation of the larger syringyl moieities such as those related to *m*/*z* 194). The 10 most important spectral intensities for the secondary type prediction occur at the *m*/*z* values of 120, 137, 154, 167, 168, 180, 181, 194, 208, and 210 (all lignin-derived).

When all the 127 features were used for the prediction of Sample ID, the spectral intensity feature with the highest importance was *m*/*z* 210 (indicative of sinapyl alcohol pyrolysate produced from syringyl moeities in lignin) irrespective of the ML algorithm used. The spectral intensities at the *m*/*z* values of 60, 120, 137, 154, 167, 168, 180, 181, 194, and 210 were among the top 10 important features for all or most of the six different types of ML algorithms used for predicting the Sample ID. Since the spectral intensity at *m*/*z* 120 (derived from coumarates particularly in grasses) is not even in the top 25 of the 127 features used for primary type prediction, for all but one of the ML algorithms used, we can safely assume that the spectral intensity at *m*/*z* 120 has little effect on the primary type, whereas it is quite important in determining the secondary type or Sample ID. The ion at *m*/*z* 120 is likely related to the abundance of coumarates and potentially some coumaryl monomers in the biomass leading to the production of 4-vinylphenol upon pyrolysis [31]. On the other hand, the spectral intensity at *m*/*z* of 208 (derived from syringylaldehyde from lignin, either native or produced) occurs within the top 15 of the 127 spectral features, which indicates that the spectral intensity of this peak is also relevant to all the three classification problems. Similarly, it seems that the spectral intensity at *m*/*z* value of 91 (phenol derived from all lignin monomers including coumaryl alcohol and also coumarates) is relevant to predicting only the primary type. However, the accuracy values of the features used for secondary type and Sample ID prediction using different ML algorithms from the Supplementary Information File S4 SIFile4_Features_all.xlsx shows *m*/*z* 91 (phenol, derived primarily from lignin as samples were extracted of metabolites) occurs in the top 15 features for both secondary type and Sample ID prediction. Thus, the spectral intensity at *m*/*z* 91 is important in the context of all the three different classifications.

As shown in Supplementary Information File S5 SIFile5_Features_corr.xlsx, the same set of 106 non-correlated features were used for the three different classification problems, although the feature importance values varied between the different classification problems and the ML algorithm used. Among the non-correlated features, the spectral intensities at *m*/*z* values of 91 (phenolics), 107 (phenolics), 137 (guaiacyl monomers) and 139 (syringyl monomers) occur consistently occur in the top 10 features in all the three classification problems for all or most of the ML classifiers. The spectral intensity at the *m*/*z* value of 135 (derived from guaiacyl monomers in lignin) is more important for primary type prediction than for the other two classifications. Similarly, the spectral peak at the *m*/*z* values of 96 and 111, derived from sugars, are more relevant to the primary and secondary types compared to the Sample ID. On the other hand, the spectral peak at the *m*/*z* value of 60, primarily derived from sugars, is most relevant to the Sample ID, while it is increasingly less relevant for the secondary and primary types.

Features sets obtained using *p*-values are provided in the Supplementary Information File S6 SIFile6_Features_pcorr.xlsx. Unlike the non-correlated features, the feature sets varied between the different classification problems, although they are the same for the different ML classifiers. The spectral features that are specific to only one classification problem or any two of them or all three of them are detailed in Table 3. 

**Figure 2:**
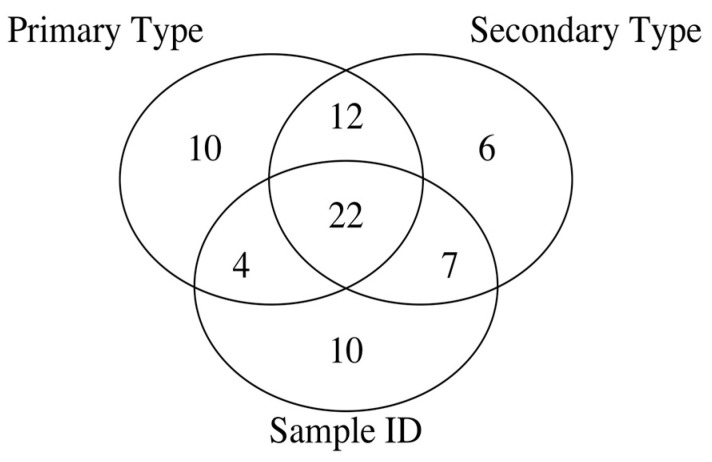
Venn diagram of py-MBMS spectral ion feature sets for different classification levels of biomass types.

Thus, the spectral intensities at the 10 *m*/*z* values, 65, 70, and 83 (primarily sugar-derived); 109, 112, 174, 177, 192, 203, and 219 (derived from various sources, typically minor contributions to spectra), are important only for the primary type classification problem, whereas the spectral intensities at the 6 *m*/*z* values of 77 (phenolics), 97 (sugars), 111, 117, 153, and 162 (various sources, minor spectral peaks) are relevant only in the context of the secondary type classification. The spectral intensities at the 10 *m*/*z* values 79 (lignin), 80 (phenolics), 100 (sugars), 106 (phenolics), 113 (sugars), 116 (sugars), 161 (hydrocarbons), 190, 211 (lignin), and 212 (lignin) are important only for the Sample ID prediction but typically occur at low intensities in the spectra. On the other hand, the spectral intensities at 22 *m*/*z* values, which are provided in the first row of Table 3, are important for all the three different classification problems and are typically abundant ions generated in biomass pyrolysis mass spectra. The Venn diagram in Figure 2 shows the overlap of the feature sets obtained via *p*-value based selection for the three different classification problems irrespective of the ML algorithm used for such classification. In general, these ions come from a variety of sources, are otherwise unannotated and/or represent fragments of hydrocarbon compounds (noting that a large portion of them are odd-numbered).

#### 2.3.2. Models Constructed from Data after RUV Correction

The removal of correlated features in RUV corrected py-MBMS spectra results in 110 instead of 127 features, whereas using *p*-value based feature selection lowers the number of features to 46, 49, and 35 for the prediction of primary type, secondary type, and Sample IDs, respectively. As in Table 2, the results in Table 4 are obtained for specific values of random number seeds that are kept invariant between the different ML classifiers to make the results reproducible. It is evident from Table 4 that although all the classifiers have comparable performance for predicting the primary and secondary types, the k-NN classifier and the decision tree classifier do not fare as well as the other classifiers in predicting the Sample IDs. Details about the features corresponding to the full feature set of 127 spectral intensities for all the three different classification problems are provided in the Supplementary Information File S7 SIFile7_Features_all_RUV.xlsx. The subset of 110 non-correlated spectral intensities, including the importance of each feature for the different ML algorithms used for the primary type, secondary type, and Sample ID classification problems are described in the Supplementary Information File S8 SIFile8_Features_corr_RUV.xlsx. The still smaller subsets of features obtained using *p*-value for all the three classifications are described in the Supplementary Information File S9 SIFile9_Features_pcorr_RUV.xlsx.

As shown in SIFile7_Features_all_RUV.xlsx for predicting the primary type using all 127 spectral features, the two most important features for five of the six ML algorithms used were the spectral intensities at the *m*/*z* values of 167 and 194 (S lignin). Spectral intensities at *m*/*z* values of 139, 154, 167, 168, 181, 194, 196, 208, and 210, all deriving from lignin, frequently occurred in the top 10 features for all or most of the six different types of ML algorithms used for predicting the primary type based on the RUV corrected spectra. In contrast to the results before RUV correction, the spectral intensities at *m*/*z* values of 91 (phenol), 107 (phenolics), and 137 (G lignin) are not as important in predicting the primary type, whereas the spectral intensity at 139 gains importance as a feature in primary type prediction. Therefore, ions 91, 107, and 137 were likely more impacted by instrumental drift. For prediction of the secondary type using all 127 spectral features, the two most important features for five of the six ML algorithms used were the spectral intensities at the *m*/*z* values of 154 and 210, which were both derived from syringyl lignin units. The spectral intensities at the *m*/*z* values of 154 (S lignin), 167 (S lignin), 168 (lignin), 180 (lignin), 181 (G lignin), 194 (S lignin), 208 (S lignin), 210 (S lignin), and 332 (S lignin) occur most frequently among the top 10 features for secondary type prediction. Thus, the spectral intensities at *m*/*z* values of 120 and 137 become less important, whereas *m*/*z* value of 332 becomes more important in predicting the secondary type after the RUV correction, which may be expected based on the principal components observed after the RUV correction. When all the 127 features were used for prediction of Sample ID, the spectral intensity feature with the highest importance was the one at *m*/*z* value of 210, and the second most important feature was the spectral intensity at *m*/*z* value of 167 irrespective of the ML algorithm used. The spectral intensities at the *m*/*z* values of 124, 137, 154, 167, 168, 180, 181, 182, 194, and 210 occur most frequently among the top 10 features for Sample ID prediction.

Similar to before RUV correction, SIFile8_Features_corr_RUV.xlsx shows the same set of 110 non-correlated features that were used for the three different classification problems, where the feature importance values varied between the different classification problems and the ML algorithm used. The two most important non-correlated features for the primary type prediction are the spectral intensities at *m*/*z* values of 139 and 154, both originating from S lignin, in particular 2,6-dimethoxyphenol, for five out of the six classifiers. The spectral intensities at *m*/*z* values of 114 (hexose sugars) and 154 (S-lignin) consistently occur among the three most important features for secondary type prediction using the non-correlated features for five out the six classifiers used. The *m*/*z* value of 332 is another important feature in the non-correlated feature set for secondary type prediction, since it occurs among the top three candidates for four out the six classifiers used. For the prediction of the Sample ID using the non-correlated features, the three most important features are the spectral intensities at *m*/*z* values of 114, 124, and 154, corresponding to three different biopolymer constituents being hexose sugars, G lignin, and S lignin, respectively. It should be noted that the spectral intensity at *m*/*z* value of 154 is consistently the most important feature in predicting the primary type, secondary type, and Sample ID for all the six classifiers used.

As shown in Supplementary Information File S8 SIFile8_Features_pcorr_RUV.xlsx, the feature sets varied between the different classification problems (primary type, secondary type, Sample ID), although they are the same for the different ML classifiers. The spectral features that are specific to only one classification problem or any two of them or all three of them based on RUV corrected data are detailed in Table 5.

The spectral intensities at the 16 *m*/*z* values, 65, 72, 74, 78, 81, 83, 98, 109, 110, 115, 161, 163, 166, 197, 205, and 296, which are typically fragment or low abundance ions with ambiguous sources, are important only for the primary type classification problem. The spectral intensities at the 11 *m*/*z* values of 68, 103, 106, 117, 122, 129, 130, 148, 153, 209, and 211 are also ambiguously assigned and are relevant only in the context of the secondary type classification using RUV corrected spectra. The spectral intensities at the 4 *m*/*z* values 55, 80, 113, and 418 (S lignin dimer) are important only for the Sample ID prediction.

**Figure 3:**
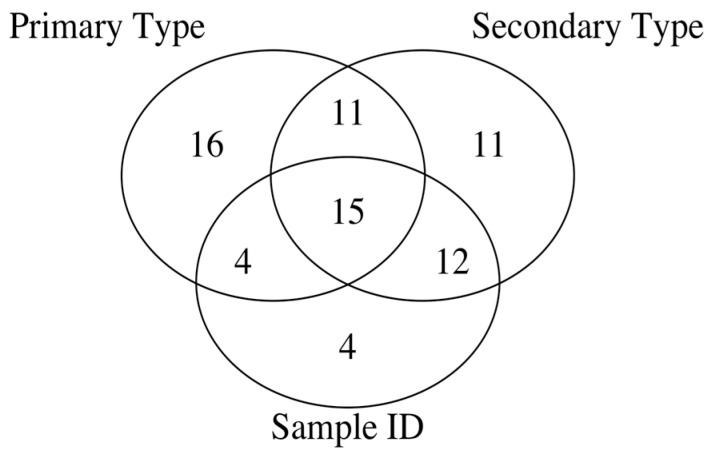
Venn diagram of py-MBMS spectral ion feature sets as RUV corrected spectra for different classification levels of biomass types.

The spectral intensities at 15 *m*/*z* values, which are provided in the first row of Table 5, are important for all the three different classification problems and make up the only grouping of ions consisting of a number of annotated or otherwise abundant ions typically observed in the spectra. The Venn diagram in Figure 3 shows the overlap of the feature sets for the three different classification problems irrespective of the ML algorithm used for such classification. The results are in contrast to the number of spectral intensities in the *p*-value-based feature set that are relevant to the three different classification problems. Comparison of the *p*-value based feature sets before and after the RUV correction indicates that there is an increase in the number of spectral intensity features relevant only to the primary type classification, (10 vs. 16), as well as in the number of spectral intensity features relevant only to the secondary type classification (6 vs. 11). On the other hand, comparison of the pre-RUV corrected versus post-RUV corrected *p*-value based feature sets indicates that there is a reduction in the number of spectral intensity features that are relevant only to the Sample ID classification problem, (10 vs. 4). Finally, the number of spectral intensities in the *p*-value based feature set that are relevant to all the three classification problems (primary type, secondary type and Sample ID) shrinks from 22 before the RUV correction to 15 after the RUV correction.

#### 2.3.3. Classification of Biomass Mixtures

ML algorithms were also used to classify between pure and binary mixtures of primary types from the RUV corrected spectral intensity data. The six possible primary types for this classification are hardwoods, softwoods, grasses, hardwoods + softwoods, hardwoods + grasses, and softwoods + grasses. In this context, it should be mentioned that the binary mixture of two different samples having the same primary type is classified as a pure primary type; for example, the binary mixture of two different samples having different secondary types and Sample IDs but the same primary type, e.g., hardwoods, is included in the hardwoods class. A total of 177 data points using 127 *m*/*z* spectral intensities were used as the largest set of features. The removal of correlated features results in 97 features, whereas using *p*-value based feature selection lowers the number of features to only 16.

Among the classifiers that work for all the feature sets, the random forest, Gaussian Naïve Bayes (GNB), and extreme gradient boosting (XGB) classifiers perform better than the k-NN and the decision tree classifiers (Table 6). Given the high accuracy and 10-fold cross-validation scores with only 177 data points, it can be expected that these classification methods can be achieved with much higher accuracies and cross-validation scores when more data points are available. For prediction of the mixtures, the *p*-value based feature selection does not work for the MLP classifier. The MLP classifier is expected to work with the minimal set of 16 features if there exists more data for training the classifier. It is evident from Supplementary File S10 SIFile10_Features_primarymix_RUV.xlsx that predicting the category of pure or binary mixture of primary types, using all 127 spectral features, the most important features for five of the six ML algorithms used was ion *m*/*z* 167 (lignin-derived). The spectral intensities at *m*/*z* 103 (pentose sugars and G lignin), 137 (G lignin), 154 (S lignin), 167 (lignin), 168 (lignin), 180 (lignin), 181 (S lignin), 182 (S lignin), 194 (S lignin), 208 (S lignin), and 210 (S lignin) frequently occurred in the top 10 features for all or most of the six different types of ML algorithms used for predicting the primary type. Table 7 provides a comparison of the most important (top 10) features for the pure primary type classification and those for the classification into pure and mixed primary types. It is evident from Table 7 that the spectral intensities at *m*/*z* values of 103, 137, 180, and 182 gain more relevance, and those at 139 (S lignin) and 196 (more prevalent in softwoods) become less relevant when classifying biomass feedstock samples into pure or binary mixtures of primary types as compared to classification of samples to pure primary types.

## 3. Discussion

Spectral drift and changes across large sample sets are a likely occurrence. Such changes result from the regular tuning of the py-MBMS units and reactivity and cleaning of condensates that accumulate in the transfer zones between pyrolysis chambers. The analysis of standards at a regular frequency are needed to maintain quality assurance. Certain spectral features may also be used as internal standard metrics to remove or account variance associated with instrumentation. Instrumental drift within sample sets (samples analyzed together within a short time frame without significant instrument changes or maintenance occurring in the same time frame) and across sets presents challenges for comparing data. Such drift also complicates the constructing models used to predict classes or quantitate characteristics such as feedstock composition across different sets. Simple averaging of data from sample replicates may not suffice, as instrumental drift may present changes to the data such that averaging would not accurately represent the characteristics of the samples analyzed. Therefore, removal of variance attributed to the instrument using standards and specific spectral features of standards was explored. The variance removal was implemented by using a method developed for normalizing RNAseq data in an attempt to improve sample comparison within and across sample sets. Since the number of spectral intensities in the *p*-value based feature sets are lower in the RUV corrected spectra, it may be deduced that RUV correction reduces the importance of ion features that are impacted by instrument drift. However, RUV correction did impact the interpretation of the data (for example, principal component ions and important features in ML classifiers) and did not necessarily improve the predictive outcomes of the ML classification.

The predictive accuracy of the ML classifiers does not significantly differ between the exclusion and the inclusion of the RUV correction of the spectral data. This observation indicates that the instrument drift in the input spectral intensities is not sizeable so far as the model predictive capability is concerned. In other words, although the RUV correction is expected to reduce the skewness of the data as well as the prominence of outliers [32], in the present case, this effect is not large enough to significantly alter the model-based prediction accuracies. In addition, the importance of particular spectral intensities or ions used as features in the ML classifiers differs slightly before and after RUV correction. After the RUV correction, the important ions are more frequently of lower abundance and have typically received less attention with respect to chemical assignments. These ions may provide overlooked insight to the structural and compositional variation that occurs within and across plant types. Such variation could be further elucidated in combination with other analytical tools. Ions that were less important to the models after the RUV classification, such as 91 and 107, are typically fragment ions with lower molecular weight. This is expected, as these ions are impacted the most by changes in the instrument. While there are other ways to remove instrument variance or variance attributed to other variables (see Harman-Ware, Macaya-Sanz et al. [28]), data interpretation should be considered by subject matter experts before and after correction to ensure data quality and proper attribution to variance. In this example, RUV correction may not have been necessarily needed for classification but did result in different interpretation of the spectral results, which may otherwise highlight the need to focus on typically neglected spectral features.

Overall, the decision tree classifier had lower accuracy of prediction compared to the other ML classifiers. Decision trees lose valuable information when handling continuous variables and perform better when predicting on the basis of categorical variables [33]. Moreover, a small change in the data can result in a large change in the structure of the decision tree yielding instability and a higher probability of overfitting [34]. It has generally been observed that decision trees exhibit a lower accuracy of prediction for a given dataset compared to other machine learning algorithms [35].

While these biomass samples vary in their primary components of lignin and sugar content, these findings indicate that the spectral differences and pyrolysis analysis methodology are not just differentiating the biomass based on the primary components relative abundances alone. The structures of the biopolymers and presence of specific sugars or other species are also driving the relative abundance of pyrolysate production and subsequent spectral variation. Ions were important for the classification of primary type, secondary type, and sample IDs without particular patterns related to their corresponding sources. However, for the mixtures, it was clear that the relative importance was the relative abundance of S- and G-lignin-derived ions as well as the ions that correspond to components typically present in conifers. We hypothesized that *m*/*z* 120 (coumarate) would be important to differentiate primary types, particularly for grasses, as coumarates are typically more abundant in grass species, but this ion is apparently not important in relation to the other ions for prediction of primary biomass types using the ML classifiers here (prior to RUV correction). The fact that Aspen, a hardwood, along with primarily S- and G- lignin derived ions, is being used for the RUV correction, could explain why *m*/*z* 120 was not a principal component with significant loadings after RUV correction and also not an important feature in the classifications. This finding highlights the importance of selecting relevant variables for correction but otherwise indicates the potential discrepancies associated with machine learning classification outcomes and hypotheses based on a priori knowledge of data structure.

## 4. Materials and Methods

### 4.1. Lignocellulosic Biomass Feedstocks

A variety of different lignocellulosic biomass reference materials obtained from NIST, from the BioEnergy Science Center/Center for Bioenergy Innovation (poplars grown in different locations from the states of OR and WA in the Pacific Northwest region, USA), ArborGen Inc., SC, USA (Eucalyptus IBP1), as well as others from the National Renewable Energy Laboratory (Golden, CO) were analyzed for use as a training set, their identities and composition profiles (determined by NREL LAPs [36,37]) are described in Section 2.3 and summarized in Table 8. Various mixtures of these reference materials of varying mass fractions were also made for modeling and prediction purposes. Each biomass sample had previously been debarked (where applicable), air dried, milled to 40 mesh, destarched, and ethanol extracted to produce samples with consistent particle size and to ensure homogeneous sampling. Aspen was analyzed in 96 technical replicates and all other samples were analyzed with 48 technical replicates.

### 4.2. Pyrolysis-Molecular Beam Mass Spectrometry (py-MBMS)

Py-MBMS was performed according to previously described methods [6,7,28,38]. First, 4 mg of dried and ground material (1 mm mesh) was pyrolyzed in a Frontier PY2020 at 500 °C for 30 s, and pyrolysis vapors were analyzed using an Extrel Super Sonic MBMS Model Max 1000 (Extrel CMS, Pittsburg, PA, USA). Lignin content has traditionally been estimated in py-MBMS analyses relative to a standard of known Klason lignin content. This standard is obtained by a summation of ion intensities of *m*/*z* 120, 124, 137, 138, 150, 152, 154, 164, 167, 168, 178, 180, 181, 182, 194, 208 and 210 in mean-normalized spectra. Relative syringyl (S) values are typically calculated by summation of ion intensities of *m*/*z* 154, 167, 168, 182, 194, 208, 210. Similarly, relative guaiacyl (G) values are often determined by summation of ion intensities of *m*/*z* 124, 137, 138, 150, 164, and 178. Then, S/G ratios can be determined by dividing the sum of S-based ions by the sum of G-based ions.

### 4.3. Spectral Data Analysis

R [29] and the Unscrambler X V.10.5 (Camo Analytics, NJ, USA) were used for exploratory data analysis and statistical analysis as described previously [28]. Basic statistical analysis (i.e., mean, variance, standard deviation), principal component analysis (PCA), and clustering methods were primarily conducted using Unscrambler X. PCA was performed using a NIPALS algorithm, approximately 7 principal components depending on convergence of data, 100 iterations with 20 random cross-validation segments, on mean-centered data.

Variance attributed to tray based on instrumental drift or other time-dependent variables was corrected on the basis of standards similarly to that described in Harman-Ware and Macaya-Sanz et al. [28] but was different in this manuscript in order to compare one set to another where each set contained the same standards. Here, correction was performed using syringyl-derived ions (S = XX), guaiacyl-derived ions (G = XX), and other lignin derived ions (L = XXX). Normalization, or correction here, utilizes negative control samples which will not vary based on the technical differences between machines. The S/G used as the spike-in controls to estimate the factors of unwanted variation within a generalized linear model (GLM) and the data are adjusted accordingly.

Machine learning algorithms were used to predict the primary type, secondary type, and the Sample ID of the biomass feedstocks from the spectral intensities corresponding to a feature set of *m*/*z* peaks or subsets thereof. Thus, these are three different classification problems that use the same set of spectral features. We examined the use of six different machine learning classifiers for these three classification problems, namely the random forest classifier, the decision tree classifier, the k-nearest neighbors (k-NN) classifier, the Gaussian Naïve Bayes (GNB) classifier, the Multi-layer perceptron classifier (MLP), and the extreme gradient boosting (XGB) classifier for these three different classification problems. The accuracy of each classification is determined using an 80:20 split of the available spectral intensity dataset for training and test data respectively, whereas the 10-fold cross-validation scores (CV scores) are determined using the entire dataset. We have compared the machine learning-based predictions both before and after the removal of instrumental variance from the spectral intensities. We started with a large set of spectral intensities consisting of 127 *m*/*z* ions of known origin and significant abundance and attempted to develop classification models using this complete set of features. However, there is significant correlation between several of these spectral intensities, and the removal of any one of two correlated spectra (Pearson correlation coefficient > 0.9) reduces the number of features to 110 or 106, depending on whether RUV correction is applied or not, with minimal effect on the accuracy of prediction by the machine learning (ML) models. The number of features can be further reduced to less than 50 by backward elimination of features using *p*-values of features in a series of surrogate linear regression models. In this approach, the different categories for each classification problem are mapped to integer values, and a linear regression model to predict these integers from the complete set of spectral intensities is created and the maximum of the feature *p*-values is evaluated. The feature with the maximum *p*-value that exceeds a threshold e.g., 0.05 is the least significant feature, which is thereby removed from the feature set, and the linear regression model is constructed again with the reduced feature set. This is repeated until there exist no features in the reduced set that have *p*-values exceeding the threshold. Irrespective of the feature set used, the importance of each feature was evaluated as the mean of the 3-fold cross-validation scores for the classification model with the feature in question as the only feature.

## 5. Conclusions

We have reported the analysis of different types of biomass and mixtures of biomass by py-MBMS and demonstrated the application of machine learning classification algorithms for the prediction of the biomass types. The approaches reported here can be used to screen or analyze large populations of biomass to characterize and identify changes and differences in biomass cell wall structure and composition as well as pyrolysis behavior for applications in agriculture, biofuel production, bioengineering, genomics, smart-breeding approaches, forensics, and to inform systems biology models. Depending on whether raw spectral data or normalized data was used, classifiers performed differently and all generally used important features generally deriving from lignin in the biomass. Mixed biomass classifications were not as accurate, which was likely due to smaller data sets, but classifiers also performed differently in comparison to classifications used for pure biomass types. The biomass cell wall structural and compositional differences were clearly captured by py-MBMS analysis, and ML algorithms were capable of predicting biomass types successfully. However, additional data will improve accuracy and predictive capabilities for broader ranges of biomass types and characteristics; and ML approaches may further elucidate pyrolysis behaviors and quantitative information regarding biomass composition. These tools can be used to make informed decisions about biomass pyrolysis spectral data variation and to help develop libraries and characterize or identify trends as well as completely unknown or outlier samples when relevant standards are not available.

## Figures and Tables

**Table 1:** Summary of biomass classification problems.

Number of	Primary Type	Secondary Type	Sample ID
Classes	3	10	16
Samples	816	816	816

**Table 2:** Comparison of machine learning (ML) algorithms for prediction of primary type, secondary type, and Sample ID from py-MBMS spectral intensities prior to removal of instrumental variance (RUV) correction.

Machine Learning Algorithm	Primary			Secondary			Sample ID		
Random Forest Classifier		Number of Features	Accuracy (CV Score)		Number of Features	Accuracy (CV Score)		Number of Features	Accuracy (CV Score)
	All features	127	1.0 (1.0)	All features	127	1.0 (1.0)	All features	127	0.98 (0.99)
	Correlated features removed	106	1.0 (1.0)	Correlated features removed	106	0.99 (0.99)	Correlated features removed	106	0.96 (0.97)
	*p*-value based feature selection	48	1.0 (1.0)	*p*-value based feature selection	47	1.0 (0.99)	*p*-value based feature selection	43	0.97 (0.96)
Decision Tree Classifier		Number of Features	Accuracy (CV Score)		Number of Features	Accuracy (CV Score)		Number of Features	Accuracy (CV Score)
	All features	127	1.0 (1.0)	All features	127	0.93 (0.95)	All features	127	0.95 (0.93)
	Correlated features removed	106	1.0 (0.99)	Correlated features removed	106	0.94 (0.93)	Correlated features removed	106	0.93 (0.90)
	*p*-value based feature selection	48	1.0 (1.0)	*p*-value based feature selection	47	0.93 (0.94)	*p*-value based feature selection	43	0.89 (0.92)
k-NN Classifier		Number of Features	Accuracy (CV Score)		Number of Features	Accuracy (CV Score)		Number of Features	Accuracy (CV Score)
	All features	127	1.0 (1.0)	All features	127	1.0 (1.0)	All features	127	0.97 (0.97)
	Correlated features removed	106	1.0 (1.0)	Correlated features removed	106	1.0 (1.0)	Correlated features removed	106	0.98 (0.97)
	*p*-value based feature selection	48	1.0 (1.0)	*p*-value based feature selection	47	1.0 (1.0)	*p*-value based feature selection	43	0.96 (0.96)
GNB Classifier		Number of Features	Accuracy (CV Score)		Number of Features	Accuracy (CV Score)		Number of Features	Accuracy (CV Score)
	All features	127	1.0 (1.0)	All features	127	0.96(0.96)	All features	127	0.98 (0.98)
	Correlated features removed	106	1.0 (1.0)	Correlated features removed	106	0.95(0.95)	Correlated features removed	106	0.97 (0.97)
	*p*-value based feature selection	48	1.0 (1.0)	*p*-value based feature selection	47	0.96(0.96)	*p*-value based feature selection	43	0.97 (0.97)
XGB Classifier		Number of Features	Accuracy (CV Score)		Number of Features	Accuracy (CV Score)		Number of Features	Accuracy (CV Score)
	All features	127	1.0 (1.0)	All features	127	0.99(0.99)	All features	127	0.98 (0.97)
	Correlated features removed	106	1.0 (1.0)	Correlated features removed	106	0.99(0.98)	Correlated features removed	106	0.94 (0.95)
	*p*-value based feature selection	48	1.0 (1.0)	*p*-value based feature selection	47	0.99(0.98)	*p*-value based feature selection	43	0.95 (0.95)
MLP Classifier		Number of Features	Accuracy (CV Score)		Number of Features	Accuracy (CV Score)		Number of Features	Accuracy (CV Score)
	All features	127	1.0 (1.0)	All features	127	0.99(0.99)	All features	127	0.97 (0.97)
	Correlated features removed	106	1.0 (1.0)	Correlated features removed	106	0.99(0.99)	Correlated features removed	106	0.97 (0.96)
	*p*-value based feature selection	48	1.0 (1.0)	*p*-value based feature selection	47	0.99(0.99)	*p*-value based feature selection	43	0.95 (0.95)

**Table 3:** Spectral features (ions) specific to classification problems using spectra before RUV correction.

Primary Type	Secondary Type	Sample ID	Counts	*m*/*z* Values
True	True	True	22	60, 84, 85, 86, 91, 93, 94, 105, 114, 123, 124, 126, 135, 136, 139, 140, 144, 165, 184, 205, 209, 302
False	True	True	7	55, 58, 64, 115, 119, 125, 131
True	False	True	4	66, 197, 200, 296
False	False	True	10	79, 80, 100, 106, 113, 116, 161, 190, 211, 212
True	True	False	12	74, 92, 99, 103, 107, 110, 121, 129, 137, 148, 149, 166
False	True	False	6	77, 97, 111, 117, 153, 162
True	False	False	10	65, 70, 83, 109, 112, 174, 177, 192, 203, 219

**Table 4:** Comparison of ML algorithms for prediction of primary type, secondary type, and Sample ID from py-MBMS spectral intensities after RUV correction of the spectra.

Machine Learning Algorithm	Primary Type			Secondary Type			Sample ID		
Random Forest Classifier		Number of Features	Accuracy (CV Score)		Number of Features	Accuracy (CV Score)		Number of Features	Accuracy (CV Score)
	All features	127	1.0 (1.0)	All features	127	0.98 (1.0)	All features	127	0.96 (0.97)
	Correlated features removed	110	1.0 (1.0)	Correlated features removed	110	0.99 (0.99)	Correlated features removed	110	0.95 (0.96)
	*p*-value based feature selection	46	1.0 (1.0)	*p*-value based feature selection	49	0.99 (0.99)	*p*-value based feature selection	35	0.95 (0.96)
Decision Tree Classifier		Number of Features	Accuracy (CV Score)		Number of Features	Accuracy (CV Score)		Number of Features	Accuracy (CV Score)
	All features	127	1.0 (1.0)	All features	127	0.98 (0.97)	All features	127	0.91 (0.88)
	Correlated features removed	110	1.0 (1.0)	Correlated features removed	110	0.97 (0.96)	Correlated features removed	110	0.90 (0.88)
	*p*-value based feature selection	46	1.0 (1.0)	*p*-value based feature selection	49	0.98 (0.96)	*p*-value based feature selection	35	0.93 (0.89)
k-NN Classifier		Number of Features	Accuracy (CV Score)		Number of Features	Accuracy (CV Score)		Number of Features	Accuracy (CV Score)
	All features	127	1.0 (1.0)	All features	127	0.99 (0.99)	All features	127	0.93 (0.93)
	Correlated features removed	110	1.0 (1.0)	Correlated features removed	110	0.98 (0.99)	Correlated features removed	110	0.93 (0.93)
	*p*-value based feature selection	46	1.0 (1.0)	*p*-value based feature selection	49	0.99 (0.98)	*p*-value based feature selection	35	0.92 (0.92)
GNB Classifier		Number of Features	Accuracy (CV Score)		Number of Features	Accuracy (CV Score)		Number of Features	Accuracy (CV Score)
	All features	127	1.0 (1.0)	All features	127	0.98 (0.98)	All features	127	0.96 (0.96)
	Correlated features removed	110	1.0 (1.0)	Correlated features removed	110	0.98 (0.97)	Correlated features removed	110	0.97 (0.96)
	*p*-value based feature selection	46	1.0 (1.0)	*p*-value based feature selection	49	0.98 (0.98)	*p*-value based feature selection	35	0.97 (0.95)
XGB Classifier		Number of Features	Accuracy (CV Score)		Number of Features	Accuracy (CV Score)		Number of Features	Accuracy (CV Score)
	All features	127	1.0 (1.0)	All features	127	0.98 (0.98)	All features	127	0.96 (0.97)
	Correlated features removed	110	1.0 (1.0)	Correlated features removed	110	0.98 (0.99)	Correlated features removed	110	0.97 (0.97)
	*p*-value based feature selection	46	1.0 (1.0)	*p*-value based feature selection	49	0.98 (0.98)	*p*-value based feature selection	35	0.96 (0.97)
MLP Classifier		Number of Features	Accuracy (CV Score)		Number of Features	Accuracy (CV Score)		Number of Features	Accuracy (CV Score)
	All features	127	1.0 (1.0)	All features	127	0.99 (1.0)	All features	127	0.96 (0.95)
	Correlated features removed	110	1.0 (1.0)	Correlated features removed	110	1.0 (0.99)	Correlated features removed	110	0.96 (0.96)
	*p*-value based feature selection	46	1.0	*p*-value based feature selection	49	0.99 (0.99)	*p*-value based feature selection	35	0.96 (0.95)

**Table 5:** Spectral features (ions) specific to classification problems using RUV-corrected data.

Primary Type	Secondary Type	Sample ID	Counts	*m*/*z* Value
True	True	True	15	73, 93, 94, 105, 107, 114, 123, 124, 126, 135, 140, 154, 162, 219, 302
False	True	True	12	58, 91, 95, 97, 100, 119, 131, 136, 139, 190, 200, 332
True	False	True	4	60, 79, 165, 203
False	False	True	4	55, 80, 113, 418
True	True	False	11	57, 66, 84, 85, 86, 92, 120, 144, 149, 174, 192
False	True	False	11	68, 103, 106, 117, 122, 129, 130, 148, 153, 209, 211
True	False	False	16	65, 72, 74, 78, 81, 83, 98, 109, 110, 115, 161, 163, 166, 197, 205, 296

**Table 6:** Comparison of ML algorithms for prediction of pure and mixed primary biomass types from py-MBMS spectral intensities after RUV correction of the spectra.

Machine Learning Algorithm	Primary Type (Pure + Binary Mixtures)		
Random Forest Classifier		Number of Features	Accuracy (CV Score)
	All features	127	0.97 (0.94)
	Correlated features removed	97	0.89 (0.93)
	*p*-value based feature selection	16	0.92 (0.89)
Decision Tree Classifier		Number of Features	Accuracy (CV Score)
	All features	127	0.86 (0.91)
	Correlated features removed	97	0.83 (0.82)
	*p*-value based feature selection	16	0.72 (0.79)
k-NN Classifier		Number of Features	Accuracy (CV Score)
	All features	127	0.92 (0.90)
	Correlated features removed	97	0.83 (0.85)
	*p*-value based feature selection	16	0.81 (0.78)
GNB Classifier		Number of Features	Accuracy (CV Score)
	All features	127	0.97 (0.94)
	Correlated features removed	97	0.97 (0.94)
	*p*-value based feature selection	16	0.92 (0.89)
XGB Classifier		Number of Features	Accuracy (CV Score)
	All features	127	0.89 (0.92)
	Correlated features removed	97	0.94 (0.90)
	*p*-value based feature selection	16	0.92 (0.87)
MLP Classifier		Number of Features	Accuracy (CV Score)
	All features	127	0.97 (0.95)
	Correlated features removed	97	0.97 (0.93)
	*p*-value based feature selection	16	0.25 (0.24)

**Table 7:** Spectral features (ions) specific to classification problems using RUV-corrected data.

Primary Type (Pure Samples)	Primary Type (Pure Samples + Binary Mixtures)	Counts	*m*/*z* Values
True	True	7	154, 167, 168, 181, 194, 208, 210
True	False	2	139, 196
False	True	4	103, 137, 180, 182

**Table 8:** Composition analysis of lignocellulosic biomass analyzed by py-MBMS.

Sample ID	Family (Primary Class)	Species (Secondary Class)	Total Ash %	% Ethanol Extractives	% Lignin	% Glucan	% Xylan	% Galactan	% Arabinan	% Mannan
BESC SWG	Grass	Switchgrass	5.23	3.68	23.90	30.97	19.18	1.79	3.33	0.00
Corn Stover L	Grass	Corn Stover	5.09	3.37	14.78	32.49	20.21	1.67	2.40	0.00
Corn Stover	Grass	Corn Stover	5.42	3.53	15.42	34.93	18.94	1.19	1.86	0.00
NIST 8491	Grass	Sugarcane Bagasse	4.00	4.40	24.20	40.20	21.50	0.60	1.80	0.40
NIST 8494	Grass	Wheat Straw	10.30	13.00	18.00	37.60	21.70	0.80	2.50	0.30
Aspen	Hardwood	*Populus tremuloides*	0.72	3.22	24.05	39.08	16.43	1.24	0.25	1.98
BESC Poplar	Hardwood	*Populus trichocarpa*	0.55	1.41	26.95	46.17	14.76	0.97	0.40	2.80
CBI Poplar	Hardwood	*Populus trichocarpa*	0.50	0.00	23.22	43.99	15.60	1.27	0.62	3.11
IBP1	Hardwood	*Eucalyptus*	0.67	1.15	29.15	39.38	13.36	1.45	0.00	2.37
NIST 8492	Hardwood	*Poplulus deltoides*	1.00	2.40	26.20	43.20	13.90	0.60	0.70	2.00
Pop 068	Hardwood	*Populus trichocarpa*	0.54	8.17	20.65	35.88	13.04	0.99	0.00	3.92
Pop 93968	Hardwood	*Populus trichocarpa*	0.52	1.42	26.26	40.61	15.38	1.18	0.00	4.21
Lob 6A1	Softwood	Loblolly Pine	0.24	1.77	33.30	37.79	6.94	2.69	0.00	13.57
Lob 6G1	Softwood	Loblolly Pine	0.24	1.81	29.13	40.75	6.03	2.16	0.00	16.00
Lob 6H2	Softwood	Loblolly Pine	0.33	1.44	31.77	41.13	6.12	1.91	0.00	16.51
NIST 8493	Softwood	Monterey Pine	0.30	2.70	26.60	42.90	6.20	2.40	1.50	10.90

## Data Availability

Data is available from authors upon request.

## Supplementary Materials

The following are available online at https://www.mdpi.com/article/10.3390/ijms22084107/s1, Supplementary File S1, SIFile1_DescStats.xlsx: Statistical analyses for the biomass experimental data. Supplementary File S2, SIFile2_PearsonCC.xlsx: Pearson correlation coefficients for RUV/instrumental drift corrected ions. Supplementary File S3, SIFile3_PCARUVFigure.pdf: Principal component analysis of py-MBMS data from different types of lignocellulosic biomass after RUV correction of spectral data. Supplementary File S4, SIFile4_Features_all.xlsx: Importance of all features (*m*/*z* peak intensities) selected from py-MBMS spectra before RUV correction for classification of biomass samples into different primary types, secondary types, and Sample IDs. Supplementary File S5, SIFile5_Features_corr.xlsx: Importance of non-correlated features (*m*/*z* peak intensities) selected from py-MBMS spectra before RUV correction for classification of biomass samples into different primary types, secondary types, and Sample IDs. Supplementary File S6, SIFile6_Features_pcorr.xlsx: Importance of *p*-value based features (*m*/*z* peak intensities) selected from py-MBMS spectra before RUV correction for the classification of biomass samples into different primary types, secondary types and Sample IDs. Supplementary File S7, SIFile7_Features_all_RUV.xlsx: Importance of all features (*m*/*z* peak intensities) selected from RUV-corrected py-MBMS spectra for classification of biomass samples into different primary types, secondary types, and Sample IDs. Supplementary File S8, SIFile8_Features_corr_RUV.xlsx: Importance of non-correlated features (*m*/*z* peak intensities) selected from RUV-corrected py-MBMS spectra for classification of biomass samples into different primary types, secondary types and Sample IDs. Supplementary File S9, SIFile9_Features_pcorr_RUV.xlsx: Importance of *p*-value based features (*m*/*z* peak intensities) selected from RUV-corrected py-MBMS spectra for classification of biomass samples into different primary types, secondary types and Sample IDs. Supplementary File S10, SIFile10_Features_primarymix_RUV.xlsx: Importance of all features (*m*/*z* peak intensities) in the RUV-corrected py-MBMS spectra for classification of biomass samples into different categories of pure or binary mixture of primary types.

## Abbreviations

ANN	Artificial neural network
DA	Discriminant analysis
GLM	Generalized linear model
GNB	Gaussian naïve Bayes
ML	Machine learning
MLP	Multi-layer perceptron
PCA	Principal component analysis
PCR	Polymerase chain reaction
PLS	Partial least squares
Py-MBMS	Pyrolysis molecular beam mass spectrometry
RF	Random forest
RNA	Ribo-nucleic acid
RUV	Remove unwanted variation
XGB	Extreme gradient boosting
MDPI	Multidisciplinary Digital Publishing Institute
NIST	National Institute of Standards and Technology
DOAJ	Directory of open access journals
ANN	Artificial neural network
DA	Discriminant analysis
GLM	Generalized linear model
GNB	Gaussian Naïve Bayes
ML	Machine learning
MLP	Multi-layer perceptron
PCA	Principal component analysis
