# Correction: Nag et al. Machine Learning-Based Classification of Lignocellulosic Biomass from Pyrolysis-Molecular Beam Mass Spectrometry Data. *Int. J. Mol. Sci.* 2021, *22*, 4107

**DOI:** 10.3390/ijms26199482

**Published:** 2025-09-28

**Authors:** Ambarish Nag, Alida Gerritsen, Crissa Doeppke, Anne E. Harman-Ware

**Affiliations:** 1 Computational Science Center, National Renewable Energy Laboratory, 15013 Denver West Pkwy, Golden, CO 80401, USA; ambarish.nag@nrel.gov (A.N.); alida.gerritsen@nrel.gov (A.G.); 2 Renewable Resources and Enabling Sciences Center, National Renewable Energy Laboratory, 15013 Denver West Pkwy, Golden, CO 80401, USA; crissa.doeppke@nrel.gov

A bug was found in our R code which warrants some minor changes in some tables, figures, and the related text in the paper.

**Error in Tables:** In the original publication [1], there were multiple related mistakes in Table 3 as published. The first row in Table 3 should have a value of 22 in the “Counts” column, instead of 21, and there should be an additional element, 60, in the “*m*/*z* Values” column. The fifth row of Table 3 should have 137 in the “*m*/*z* Values” column instead of 60. The asterisk next to the 91 *m*/*z* value in the first row of Table 3, along with the asterisk in the Table 3 header and the text following it, should be removed. The sentence that immediately precedes Table 3 should be removed as well. The corrected version of Table 3 is provided below.

In the original publication, there were a couple of related mistakes in Table 5. The first row in Table 5 should have a value of 15 in the “Counts” column, instead of 14, and there should be an additional element, 154, in the “*m*/*z* Values” column. The corrected version of Table 5 is provided below.

**Errors in Figures:** In the original publication, there were minor mistakes in Figures 2 and 3. In the original publication, the intersection of all three sets—primary type, secondary type, and Sample ID, has 21 and 14 elements in Figure 2 and Figure 3, respectively. The corrected Figure 2 and Figure 3, provided below, have been updated to show that the intersection of all three sets—primary type, secondary type, and Sample ID, has 22 elements in Figure 2 and 15 elements in Figure 3, respectively.

**Text Correction:** There were the following errors in the original publication. The sentence “On the other hand, the spectral intensities at 21 *m*/*z* values, which are provided in the first row of Table 3, are important for all the three different classification problems and are typically abundant ions generated in biomass pyrolysis mass spectra.” in Section 2.3.1 on Page 9 of 22, in the last paragraph, refers to an incorrect number (21) of *m*/*z* values. A correction has been made to this sentence so that the updated sentence now reads as “On the other hand, the spectral intensities at 22 *m*/*z* values, which are provided in the first row of Table 3, are important for all the three different classification problems and are typically abundant ions generated in biomass pyrolysis mass spectra.”

The sentence “The spectral intensities at 14 *m*/*z* values, which are provided in the first row of Table 5, are important for all the three different classification problems and make up the only grouping of ions consisting of a number of annotated or otherwise abundant ions typically observed in the spectra.” in Section 2.3.2 on Page 13 of 22, in the last paragraph, also mentions in incorrect number (14) of *m*/*z* values. A correction has been made to this sentence so that the updated sentence now reads as “The spectral intensities at 15 *m*/*z* values, which are provided in the first row of Table 5, are important for all the three different classification problems and make up the only grouping of ions consisting of a number of annotated or otherwise abundant ions typically observed in the spectra.” 

The sentence “Comparison of the *p*-value based feature sets before and after the RUV correction indicates that there are fewer spectral intensity features relevant only to the primary type classification (10 vs. 16) and only to the secondary type classification (6 vs. 11).” in Section 2.3.2 on Page 13 of 22, in the last paragraph, contains an incorrect word—“fewer”. This sentence is updated to “Comparison of the *p*-value based feature sets before and after the RUV correction indicates that there is an increase in the number of spectral intensity features relevant only to the primary type classification, (10 vs. 16), as well as in the number of spectral intensity features relevant only to the secondary type classification (6 vs. 11).”

The sentence “On the other hand, after the RUV correction, there is a reduction in the number of spectral intensity features in the *p*-value based feature set that are relevant only to the Sample ID classification problem (4 vs. 10) compared to the pre-RUV correction *p*-value based feature set.” in Section 2.3.2 on Page 13 of 22, in the last paragraph, contains an incorrect phrase “(4 vs. 10)”. This sentence is corrected to “On the other hand, comparison of the pre-RUV corrected versus post-RUV corrected *p*-value based feature sets indicates that there is a reduction in the number of spectral intensity features that are relevant only to the Sample ID classification problem, (10 vs. 4)”.

The sentence “Finally, the number of spectral intensities in the *p*-value based feature set that are relevant to all the three classification problems (primary type, secondary type and Sample ID) shrinks from 21 before the RUV correction to 14 after the RUV correction.” in Section 2.3.2 on Page 14 of 22, in the first paragraph, contains two incorrect values. This sentence is rectified to “Finally, the number of spectral intensities in the *p*-value based feature set that are relevant to all the three classification problems (primary type, secondary type and Sample ID) shrinks from 22 before the RUV correction to 15 after the RUV correction.”

The authors state that the scientific conclusions are unaffected. This correction was approved by the Academic Editor. The original publication has also been updated.

## Figures and Tables

**Figure 2:**
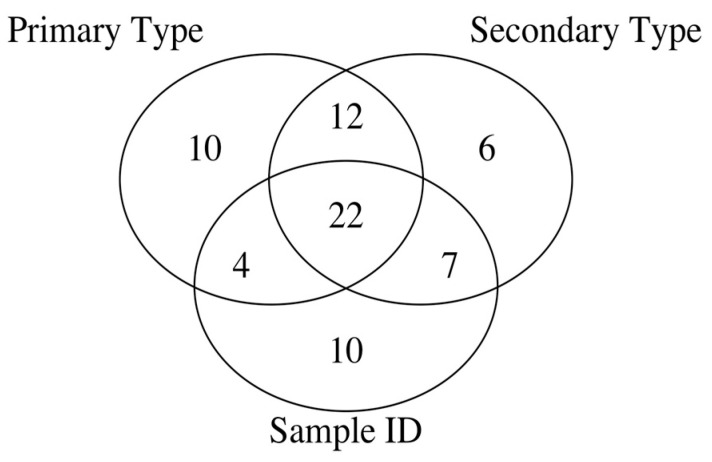
Venn diagram of py-MBMS spectral ion feature sets for different classification levels of biomass types.

**Figure 3:**
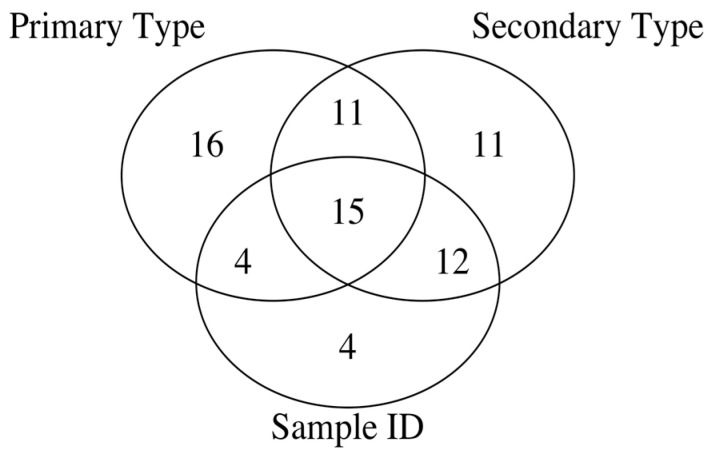
Venn diagram of py-MBMS spectral ion feature sets as RUV corrected spectra for different classification levels of biomass types.

**Table 3:** Spectral features (ions) specific to classification problems using spectra before RUV correction.

Primary Type	Secondary Type	Sample ID	Counts	*m*/*z* Values
True	True	True	22	60, 84, 85, 86, 91, 93, 94, 105, 114, 123, 124, 126, 135, 136, 139, 140, 144, 165, 184, 205, 209, 302
False	True	True	7	55, 58, 64, 115, 119, 125, 131
True	False	True	4	66, 197, 200, 296
False	False	True	10	79, 80, 100, 106, 113, 116, 161, 190, 211, 212
True	True	False	12	74, 92, 99, 103, 107, 110, 121, 129, 137, 148, 149, 166
False	True	False	6	77, 97, 111, 117, 153, 162
True	False	False	10	65, 70, 83, 109, 112, 174, 177, 192, 203, 219

**Table 5:** Spectral features (ions) specific to classification problems using RUV-corrected data.

Primary Type	Secondary Type	Sample ID	Counts	*m*/*z* Value
True	True	True	15	73, 93, 94, 105, 107, 114, 123, 124, 126, 135, 140, 154, 162, 219, 302
False	True	True	12	58, 91, 95, 97, 100, 119, 131, 136, 139, 190, 200, 332
True	False	True	4	60, 79, 165, 203
False	False	True	4	55, 80, 113, 418
True	True	False	11	57, 66, 84, 85, 86, 92, 120, 144, 149, 174, 192
False	True	False	11	68, 103, 106, 117, 122, 129, 130, 148, 153, 209, 211
True	False	False	16	65, 72, 74, 78, 81, 83, 98, 109, 110, 115, 161, 163, 166, 197, 205, 296
