# Supplementary figures and images for: Machine Learning-Based Classification of Lignocellulosic Biomass from Pyrolysis-Molecular Beam Mass Spectrometry Data
Source: Int J Mol Sci. 2021 Apr 15;22(8):4107. doi: 10.3390/ijms22084107 (PMC8071563; doi:10.3390/ijms22084107)

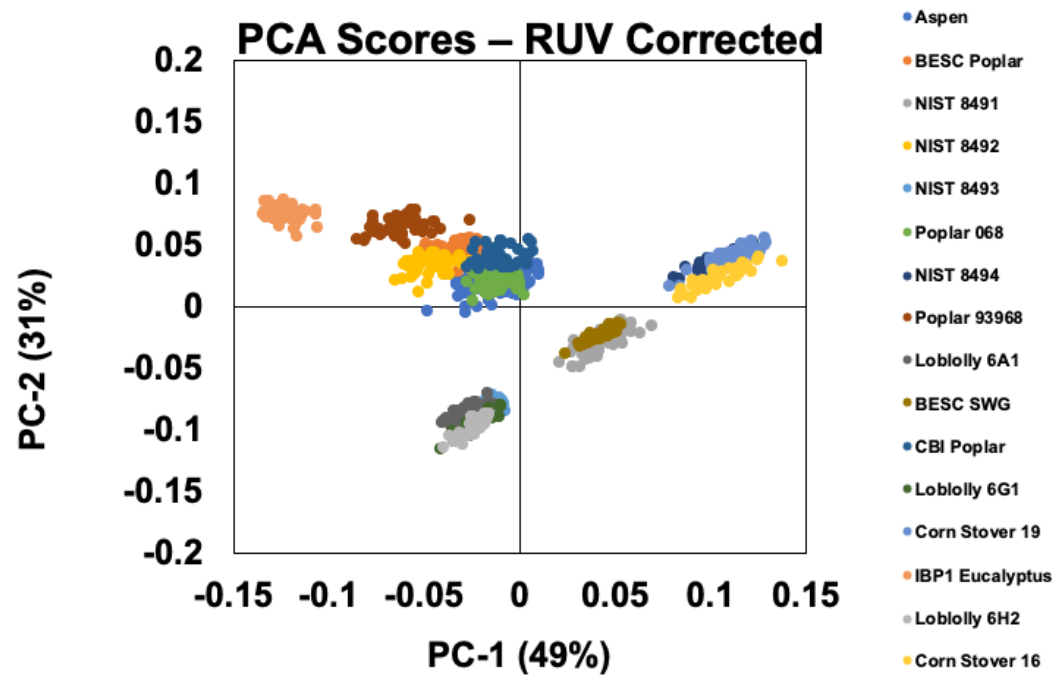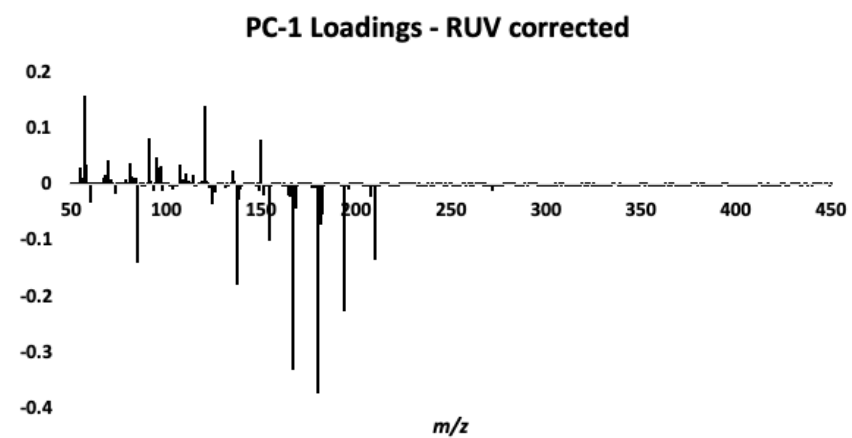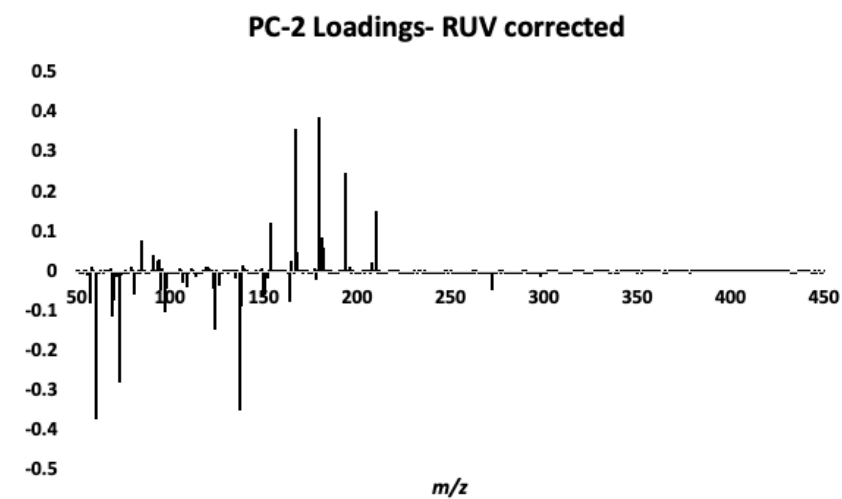

Supplement: Supplementary file 1 [file ijms-22-04107-s001.zip › SupplementaryFiles/SIFile3_PCARUVFigure.pdf]
